# Management of sentinel node re-mapping in patients who have second or recurrent breast cancer and had previous axillary procedures

**DOI:** 10.1186/1477-7819-12-205

**Published:** 2014-07-12

**Authors:** Handan Tokmak, Kerim Kaban, Mahmut Muslumanoglu, Meral Demirel, Sukru Aktan

**Affiliations:** 1 Nuclear Medicine and Molecular Imaging Department, American Hospital, Guzelbahce Sok. No: 20 Nisantasi, Istanbul 34365, Turkey; 2 Medical Oncology Department, American Hospital, Guzelbahce Sok. No:20 Nisantasi, Istanbul 34365, Turkey; 3 General Surgery Department, Istanbul University Istanbul Medical Faculty Fatih /Çapa, Istanbul 34093, Turkey; 4 General Surgery Department, American Hospital, Guzelbahce Sok. No: 20 Nisantasi, Istanbul 34365, Turkey

## Abstract

**Background:**

In patients with recurrent or second primary ipsilateral breast cancer, axillary staging is the key factor in locoregional control and a strong prognostic characteristic. The efficient evaluation of lymphatic drainage of re-sentinel lymph node biopsies (re-SLNBs) has remained a challenge in the management of ipsilateral primary or recurrent breast cancer patients who are clinically lymph node negative. This study explores whether a SLNB for patients with primary or recurrent breast cancer is possible after previous axillary surgery. It evaluates potential reasons for mapping failure that might be associated with patients in this group.

**Methods:**

Between March 2006 and November 2013, 458 patients were subjected to a breast SLNB. A lymphoscintigraphy procedure was performed on 330 patients for sentinel lymph node (SLN) mapping on the day of surgery. Seven patients with either a second primary cancer in the same breast or recurrent breast cancer were described. Two of these seven patients had axillary lymph node dissection (ALND) during previous treatments and five had SLNB. A dual mapping method was used for all patients. Preoperative lymphoscintigraphy was performed four hours before surgery.

**Results:**

SLNs were successfully remapped in six of seven (85.7%) patients, of whom five (71.43%) had previously undergone SLNB and two (28.57%) previous ALND. Localizations of SLNs were ipsilateral axillary in three patients, ipsilateral internal mammary in one patient, and contralateral axillary in two patients. An altered distribution of lymph nodes was discovered in both patients with previous ALND. In one of the two patients, metastases were found in an aberrant lymph drainage basin at the location of a non-ipsilateral axillary node (contralateral axillary SLN). The second previously ALND patient had an internal mammary SLN. In one patient, mapping was unsuccessful and the SLN was not identified.

**Conclusions:**

Altered lymphatic drainage incidence increases following breast-conserving surgery for an initial breast cancer, and the location of SLNs becomes unpredictable at the time of a second primary or recurrent ipsilateral breast cancer. This leads to the necessity of using a radionuclide (lymphoscintigraphy) for a successful re-mapping procedure. A re-SLNB is precise and beneficial even though there are few patients. A lymphoscintigraphy can identify SLNs at their new unpredicted location.

## Background

In the management of breast cancer, a sentinel lymph node biopsy (SLNB) has become standard care for staging axilla in breast cancer patients with clinically negative axillary lymph nodes [1]. The SLNB technique is a highly selective approach based on the finding that tumor cells migrating from a primary tumor metastasize to one or a few lymph nodes before involving other lymph nodes.

It has been proven that prediction of the status of surviving regional nodes can be accurately carried out from the results for sentinel lymph nodes (SLNs) [2,3]. It has been stated that the local breast cancer recurrence rate is up to 5 to 10% for patients who are having breast-conserving surgery [4,5]. In addition, new primary breast cancer connected to earlier occurrences of SLNB or previous axillary operations may be detected within the follow-up period [6].

The incidence of second primary ipsilateral or recurrent breast cancer is progressively increasing in patients with previously treated breast cancer, as would be expected. Due to the cumulative adoption of breast-conserving surgery, improved prognosis and gains in life expectancy for patients with an initial early-stage breast cancer, this clinical issue may become more common [6-10].

## Methods

**Figure 1:**
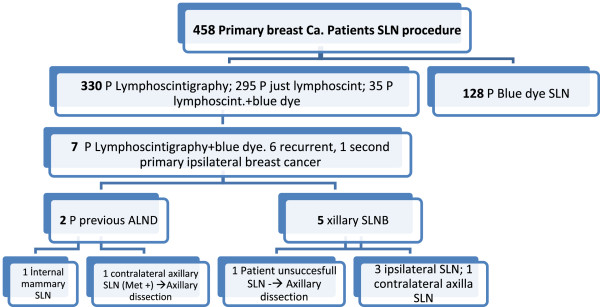
**SLN scintigraphy procedures performed and patient distribution.** ALND, axillary lymph node dissection; Ca, cancer; lymphoscint, lymphoscintigraphy; P, patients; SLN, sentinel lymph node; SLNB, sentinel lymph node biopsy. Met+: Metastases positive.

Between March 2006 and December 2013, a cohort of 458 consecutive patients with breast cancer proven by a biopsy underwent SLNB (only blue dye, blue dye plus lymphoscintigraphy, and only lymphoscintigraphy) (Figure 1). On the day of surgery, one-day lymphoscintigraphy (with or without blue dye) was performed for 330 patients (Table 1). A hand-held gamma probe (Navigator GPS, RMD Instruments, England, UK) was used to explore the SLN in the operation. A total of seven patients with a second primary cancer in the same breast or recurrent breast cancer were described in this group. Among these seven patients who already had undergone axillary procedures, two out of seven patients had axillary lymph node dissection (ALND), and five out of seven had SLNB.

**Table 1:** Characteristics of patients on whom SLN scintigraphy procedures were performed

**Characteristics of patients**		**Percentage (%)**
Number of patients	330	
Age of initial cancer diagnosis (years)	47.88	
Median age (range)	48	
Tumor size (mean size range) (mm)	18	
**Histology**		
Invasive ductal cancer	250	75.7
Invasive lobular cancer	56	16.9
Others	24	7.2
**Stage**		
Stage I	145	43.9
Stage II	148	44.8
Stage III	37	11.2
**Hormone status of tumor**		
Hormone receptor positive		
ER+	244	74
PR+	211	64
Hormone receptor negative (ER-, PR-)	79	24.80
Triple negative (ER-, PR-, HER2-)	53	16

### Sentinel lymph node biopsy technique

SLN scintigraphy procedures were performed by a nuclear medicine physician using a standard technique. All patients underwent preoperative lymphoscintigraphy on the day of surgery. A combined periareolar intradermal (the same quadrant as the tumor location) and peritumoral technique was used for all patients. After an injection of 800 to 1,000 μCi of filtered technetium sulfur colloid, dynamic and static planar images were obtained. A hand-held gamma probe (Navigator GPS) was used for identifying the SLN and to determine if there was any non-SLN. Isosulfan blue (5 mL) was injected just before the operation for 70% of all patients to detect SLN in this study. All SLNs and non-SLNs were evaluated intra-operatively by touch preparation cytology. Axillary dissection was performed if there were metastases and were identified in the SLN either intra-operatively or through permanent cytology evaluation. Regardless of SLN size, all dissected SLNs were sectioned into 2-mm thicknesses (as closely as possible). If the SLN was visually positive, less sectioning was performed (and sometimes only one section was performed). All dissected lymph nodes were evaluated through permanent cytology using hematoxylin and eosin staining and immunohistochemistry. Micro-metastases were defined as metastases ranging from 0.2 to 2 mm in size. Metastases larger than 2 mm were identified as macro-metastases. ALND was performed for the patients whose SLNB pathology results were positive. The results of the pathology were compared and evaluated with the nodes in the internal mammary areas and in axillary areas, including the contralateral axillary areas.

## Results

All patients underwent an SLNB for both their first and second axillary evaluations. SLNs were successfully remapped for six out of seven (85.7%) patients. A mean of 1.4 (range 1 to 3) lymph nodes were determined through lymphatic re-mapping for six out of seven patients. Three patients (out of seven, 42.8%) showed alternative lymphatic pathways; one of the three had an ipsilateral internal mammary node and the other two had crossed lymphatics to a contralateral axillary node. According to an actual meta-analysis of the literature (by Maaskant-Braat *et al*.) [7], which includes all studies on repeat sentinel node biopsy in patients with locally recurrent breast cancer, aberrant drainage pathways were visualized (43.2%).

One of the six successfully remapped patients had a contralateral axillary SLN, which proved to be micro-metastatic (0.3 mm in permanent pathology) (16%) and a confirmation contralateral ALND in this patient identified no additional positive axillary lymph nodes. This patient had a previous ALND (46 months previous). A pathologic examination of the internal mammary sentinel node was negative for metastases. The SLN could not be found in one patient. A complete ALND was performed in this patient. None of the dissected 11 lymph nodes from this patient were positive for metastases (false negative rate 0%).

The aforementioned patient underwent primary lymphatic mapping and then a lumpectomy, following by radiation therapy. It could be speculated that secondary inflammatory changes associated with primary radiation therapy decrease the feasibility of re-operative SLNB. In the systematic review and meta-analysis of the literature by Maaskant-Braat *et al*., sentinel node identification was successful in 452 of 692 patients (65.3%). The identification rate was significantly lower in patients who had undergone a previous ALND (52.2%) (*P* < 0.0001) compared to patients who had undergone a previous SLNB (81%) [7].

The lymphoscintigraphy procedure was performed for 330 patients for SLN mapping, and these characteristics are described in Table 1. There are other sub-classifications of breast cancer as well, such as the one that classifies breast cancers into luminal A, luminal B, basal and HER2 enriched [11]. Irrespective of the underlying breast cancer subtype, the presence of axillary lymph node metastases is associated with considerable poor disease-free as well as overall survival [12,13]. Lymph node metastases remain a very important prognostic variable, and identification of lymph node metastases can potentially help in early intervention by reducing the chances of breast cancer progression.

At a 27-month mean follow-up after the second SLNB, there were no axillary or other alternative lymph node area recurrences. There was an 85.7% success rate in patients with new or recurrent cancer in the breast who had both a previous SLNB and remapped SLNs. This is a similar success rate as the primary SLNB. In this study, re-operative SLNB failed in one out of seven patients (14.2%) (Table 2).

**Table 2:** Characteristics of patients for whom re-SLN scintigraphy procedures were performed

**Characteristics of patients**	**Number**
Number of patients	7
Age initial cancer diagnosis (years)	52.1
Median age (range )	49
**Histology**	
Invasive ductal cancer	4
Invasive lobular cancer + invasive ductal	1
Invasive lobular cancer	1
Other (mucinous cancer)	1
**Stage**	
I	3
II	2
III	2
**Sentinel lymph node re-mapping results**	
Axillary region lymph nodes	3
Other than axillary region	2
**Previous surgery**	
Breast-conserving surgery + ALND	2
Breast-conserving surgery + SLND	5

## Discussion

Reconstitution of alternative routes of drainage from the lymph nodes may lead to the undesired result of additional and previously unaffected nodes receiving primary drainage from the vicinity of the cancer-infected breast. Unpredicted alternative lymphatic pathways might be prompted by radiotherapy or previous operations could lead to damage to the usual draining lymphatics [14-16]. The high identification rate of altered lymphatic drainage in our series is attributed to previous ALND and radiotherapy (one of seven patients had ALND plus radiotherapy, one patient ALND and one patient axillary radiotherapy). There is clearly a necessity to conduct a second lymphatic mapping injection and lymphoscintigraphy before SLNB. Even patients with a virgin axilla will not have easy-to-predict patterns of drainage, and there is a greater possibility of locating nodes outside the ipsilateral axilla among patients who have underwent a previous axillary operation [17-21].

Haagensen *et al*. hypothesized that, by permeating the deep lymphatic plexus of the wall of the chest, tumor cells might disperse to the contralateral axillary [22]. In the present study, two out of 330 (0.6%) consecutive patients were identified with contralateral axillary drainage on lymphoscintigraphy. A contralateral SLN biopsy was attempted in both patients; only one of the two patients who had a contralateral axillary SLN proved to be positive for a tumor. The second patient also had an ipsilateral SLN, and both ipsilateral and contralateral SLNBs showed no metastatic involvement. There has been no ipsilateral or contralateral axillary recurrence (mean 54 months) following a negative SLNB in these patients. Contralateral axillary lymph node metastases are generally associated with the aggressiveness of the primary tumor’s pathology. Morcos *et al*. Compared data for 401 breast cancer patients who did not have contralateral axillary lymph node metastases with that of 21 patients with contralateral axillary lymph node metastases. Their retrospective analysis showed that tumor grade, lymphovascular invasion, tumor size, hormone receptor negativity and HER2 overexpression increases the risk of contralateral axillary metastases [23]. In our series, the patient with contralateral metastases had grade 2 invasive ductal carcinoma, T2, ER-PR receptor positive, and HER2 negative. As seen in our series, the histopathological features of the tumor in this patient with contralateral axillary metastasis were not aggressive. In comparison, the findings for this patient drive attention to the range of different etiologies that might have caused contralateral axillary drainage and altered the metastases area. Contralateral axillary metastases have been regarded as a distant metastatic disease, and therefore it was suggested to be treated with systemic therapy (either hormonal or chemotherapy). Emerging data indicate, that rather than a hematogeneous metastasis, the alteration of lymphatic drainage might have the pivotal role in the contralateral axillary lymphatic metastases, to this area. In addition, rarely native breast and axilla might have alternated lymphatic drainage and should be determined. As many studies show, contralateral axillary metastases and primary breast cancer could be discovered either at the same time or after having received treatment for recurring breast cancer [21-24]. Although more data needs to be gathered, a treatment approach for patients who have contralateral axillary metastasis without distant metastases should be curative in intent.

Therefore, synchronous or metachronous contralateral axillary lymph nodes without systemic metastases could be thought of as a curative disease due to the fact that they are dispersalis lymphogenic and not hematogenous. Despite a lack of consensus, patients seek this type of treatment in the hope of being cured [23-28].

There is great optimism within the scientific literature about the reliability of the capacity of re-operative SLNB to determine whether or not there are axillary nodes that test positive for metastasis [29-36]. Positive SLNs were discovered in one out of seven (14.2%) patients of our series (Table 1). After a mean 27-month follow-up period, no local axillary recurrences have been found in any patients.

## Conclusions

The present study draws attention to the increased probability of altered lymphatic drainage, resulting in new nodes being found in sites other than the ipsilateral axilla in patients who have had previous radiotherapy or previous operations. Because the altered lymphatic drainage can be detected only by lymphoscintigraphy, we suggest a lymphatic mapping injection followed by lymphoscintigraphy to identify the SLN in patients who have new or recurrent breast cancer and previous procedures (SLND or ALND), rather than proceeding directly to an axillary dissection. It should be kept in mind that if ALND was done or axillary radiotherapy carried out, it is highly unlikely that the SLN will be found in the axilla. SLN mapping with a radiocolloid is essential.

Our findings are consistent with prior studies that imply re-operative SLNB is feasible, highly useful and may provide a distinct advantage by creating an alternative to ALND for breast cancer patients (whether new or reoccurring cancer) who have had a previous axillary operation or SLNB [37,38]. A lymphoscintigraphy allows identification of the sentinel nodes, whether or not they pathologically involve cancer cells (represented by those regional nodes with a negative predictive value of almost definite), at their new unpredicted location. Further studies with larger sample sizes and longer follow-up data would be required to determine fully the statistical significance of operative lymphatic re-mapping.

## Abbreviations

ALND: axillary lymph node dissection; SLN: sentinel lymph node; SLNB: sentinel lymph node biopsy.

## Competing interests

The authors declare that they have no competing interests.

## Authors’ contributions

HT carried out the acquisition, analysis and interpretation of data, drafting of manuscript. All authors (HT, KK, MM, MD, ŞA) equally participated to critical revision. All authors read and approved the final manuscript.
